# The dynamic behavior of storage organelles in developing cereal seeds and its impact on the production of recombinant proteins

**DOI:** 10.3389/fpls.2014.00439

**Published:** 2014-09-03

**Authors:** Elsa Arcalis, Verena Ibl, Jenny Peters, Stanislav Melnik, Eva Stoger

**Affiliations:** Department of Applied Genetics and Cell Biology, University of Natural Resources and Life SciencesVienna, Austria

**Keywords:** endosperm, molecular farming, recombinant glycoproteins, cereal biotechnology, storage organelles

## Abstract

Cereal endosperm is a highly differentiated tissue containing specialized organelles for the accumulation of storage proteins, which are ultimately deposited either within protein bodies derived from the endoplasmic reticulum, or in protein storage vacuoles (PSVs). During seed maturation endosperm cells undergo a rapid sequence of developmental changes, including extensive reorganization and rearrangement of the endomembrane system and protein transport via several developmentally regulated trafficking routes. Storage organelles have been characterized in great detail by the histochemical analysis of fixed immature tissue samples. More recently, *in vivo* imaging and the use of tonoplast markers and fluorescent organelle tracers have provided further insight into the dynamic morphology of PSVs in different cell layers of the developing endosperm. This is relevant for biotechnological applications in the area of molecular farming because seed storage organelles in different cereal crops offer alternative subcellular destinations for the deposition of recombinant proteins that can reduce proteolytic degradation, allow control over glycan structures and increase the efficacy of oral delivery. We discuss how the specialized architecture and developmental changes of the endomembrane system in endosperm cells may influence the subcellular fate and post-translational modification of recombinant glycoproteins in different cereal species.

## INTRODUCTION

The endosperm is a short-lived tissue adapted for nutrient storage, which is found in both dicotyledonous and monocotyledonous seeds. However, its relative contribution to the mass of the mature seed varies greatly in different species. In cereals, the endosperm occupies the major part of the seed and consists of starchy inner endosperm cells, which are dead at the time of full maturation, and one or several outer layers of living aleurone cells, which secrete the enzymes needed to break down reserves following germination (Young and Gallie, 2000).

The endomembrane system of the developing cereal endosperm is clearly influenced by its functional specialization. Storage protein synthesis requires a highly active and well-developed endoplasmic reticulum (ER), and different types of storage organelles are rapidly formed during endosperm development to accommodate various types of storage proteins (Muntz, 1998; Shewry and Halford, 2002). ER-derived protein bodies mostly contain prolamin aggregates, whereas protein storage vacuoles (PSVs) tend to accumulate different storage protein classes, typically in separate phases, and often incorporate the content of ER-derived storage bodies (Shewry et al., 1995; Galili, 2004; Tosi et al., 2009). At the peak of storage protein production, programmed cell death occurs in the inner endosperm to generate a starchy endosperm core. In the surrounding aleurone cells, PSVs are converted into lytic vacuoles as germination commences (Bethke et al., 1998) and programmed cell death is delayed until a few days later.

To accommodate this sequence of intracellular changes in developing and germinating seeds, the endomembrane system must be extremely flexible and capable of rapid morphological and functional adaptation. Seed development and germination are therefore ideal platforms to study endomembrane plasticity and reshaping, and to follow the transition from PSV to lytic vacuole, representing a shift between resource accumulation and redistribution.

Techniques that allow the real-time visualization of membranes and organelle markers within the endosperm tissue are particularly suitable for this type of analysis because they provide insight into membrane dynamics during seed development and germination. For example, several transgenic cereal lines are now available expressing fluorescent subcellular markers and tagged or mutated storage proteins (Mohanty et al., 2009; Onda et al., 2009; Tosi et al., 2009; Ibl et al., 2014; Oszvald et al., 2014) and other fluorescent probes have been developed that can track organelles in living cells and report the subcellular organization and dynamics *in vivo* (Landrum et al., 2010). These techniques can be combined with transcriptional analysis and proteomics, which help to identify the molecular regulators for storage organelle development in cereals (Walley et al., 2013).

The mechanisms of protein trafficking and deposition in cereal endosperm can also be investigated using recombinant proteins, particularly glycoproteins (Stoger et al., 2005a; He et al., 2012; Wakasa and Takaiwa, 2013). Seeds are an attractive platform for the production of high-value recombinant pharmaceutical proteins, an approach known as molecular farming, because the specialized storage organelles allow such proteins to accumulate to high concentrations in small-volume compartments that contain molecular chaperones and disulfide isomerases to facilitate protein folding and maintain stability (De Jaeger et al., 2002; Stoger et al., 2005a; Ramessar et al., 2008a). This supportive intracellular environment means that the recombinant proteins are inert, and can be stored for long periods of time in the form of dry seeds without losing stability or activity. The accumulation of inert proteins in seeds also allows the expression of toxic proteins because these are inactive and are not expressed in vegetative tissues and therefore they do not impact on plant growth and development. The accumulation of proteins is also boosted by the triploid genome and the several rounds of endoreduplication that occur in the endosperm, thus increasing the effective number of transgene copies (Sabelli and Larkins, 2009). On this basis, many recombinant proteins have been produced successfully in cereal seeds, including commercial products such as lactoferrin, lysozyme, and human serum albumin (HSA) produced in rice by Ventria Bioscience (Fort Collins, CO, USA; Ramessar et al., 2008b; He et al., 2011b) and human growth factor for cosmetic use produced in barley by ORF Genetics (Iceland; Erlendsson et al., 2010).

Storage organelles in cereal seeds provide a clear advantage for the production of recombinant proteins, but the plasticity and remodeling of endomembrane organelles affect the accumulation and modification of proteins and so a better understanding of species-dependent and development-specific factors is needed to optimize cereal seeds for molecular farming applications. In this review article, we summarize the key features of the endomembrane organelles in major cereal species, and address the issues of cargo trafficking to these organelles in the developing endosperm of cereal species, endomembrane reshaping, and vacuolar transition during development and germination. Finally, we discuss how these endosperm-specific features affect the production of recombinant glycoproteins in plants.

## STORAGE ORGANELLES AND ENDOMEMBRANE TRAFFIC IN DIFFERENT CEREAL SPECIES

**FIGURE 1:**
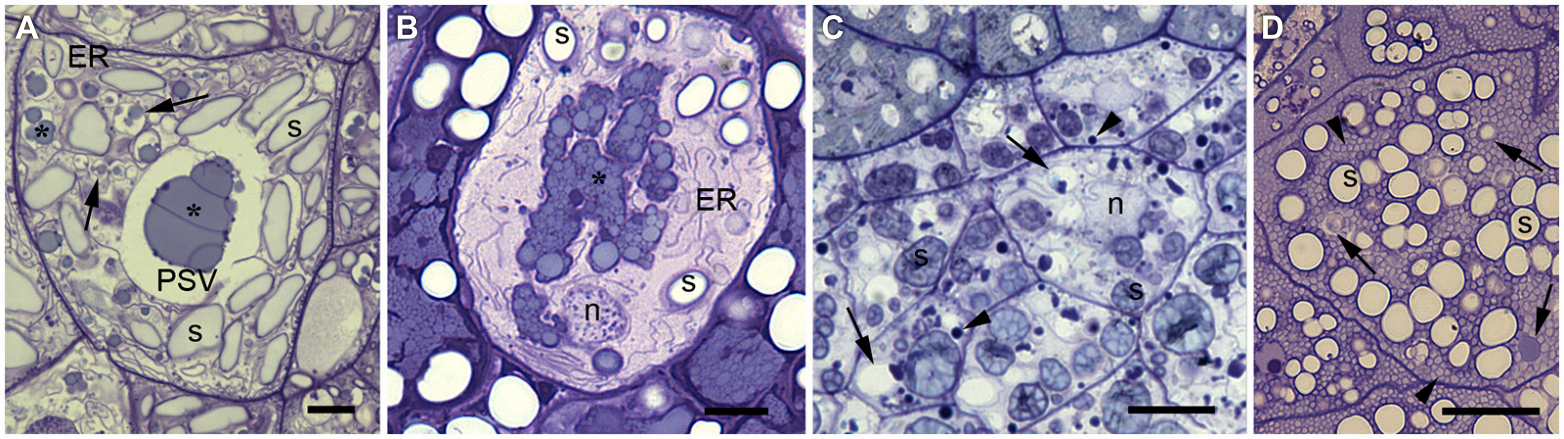
**Comparison of endosperm cells from the subaleurone layer at mid-maturation stage in wheat **(A)**, barley **(B)**, rice **(C)**, and maize **(D)****. Semithin sections (1 μm in thickness), stained with toluidine blue for light microscopy (Arcalis et al., 2004). **(A)** Wheat endosperm cells show a predominant, large central PSV. Note the huge prolamin aggregates stained in light blue (*) and the scarce globulin (triticin) bodies stained in dark blue at the periphery of the prolamins. Several smaller vacuolar compartments containing prolamins can also be observed (arrows), and well developed endoplasmic reticulum (ER) and abundant spindle like starch grains (s). **(B)** Barley endosperm cells show abundant protein deposits forming a multiphasic protein body (see the different shades of blue), habitually within a PSV. Note the abundant ER, the spheroidal starch grains (s) and an apoptotic nucleus (n). **(C)** Rice endosperm cells are the smallest depicted in this figure. Rice endosperm accumulates mainly glutelins in protein storage vacuoles (arrows). Note the abundant PSVs containing a blue stained inclusion, normally close to the tonoplast and the spherical, densely packed prolamin bodies or PB-I (arrowheads). Starch (s). **(D)** Maize endosperm stores mainly prolamins in ER-derived protein bodies (zein bodies). Zein bodies (arrowheads) reach a diameter of 1 μm, are very abundant and appear evenly spread within the cytoplasm. Several storage vacuoles can be also observed (arrows) either completely filled or with peripherical protein deposits. Starch grains (s) are spheroidal and similar in size to the PSVs. Scale bar equals 10 μm.

The endomembrane system in the developing cereal endosperm is highly specialized to accommodate different storage proteins, and differences are observed between cereal species. A representative endosperm cell from each of the four major cereals is shown in **Figure 1**. The vacuolar storage compartment can be identified easily in wheat (**Figure 1A**) and barley (**Figure 1B**) because large prolamin deposits are found in a central vacuole. In contrast, rice and maize endosperm cells do not have a single prominent protein storage site. Rice endosperm cells uniquely separate globulins and prolamins into separate compartments: globulins are sorted into numerous small PSVs whereas prolamins accumulate in ER-derived protein bodies (**Figure 1C**). Both compartments are evenly distributed within the endosperm cell cytoplasm. Maize, like wheat and barley, predominantly stores prolamins, which in this species are known as zeins. These form highly conserved protein bodies derived from the ER (Lending et al., 1989) and fill up the cytoplasm of endosperm cells together with a small number of vacuolar compartments that accumulate globulins (Woo et al., 2001; Arcalis et al., 2010; **Figure 1D**).

### SECRETION VS. STORAGE IN ENDOSPERM CELLS

The endosperm tissue of many plants is composed of uniform, living reserve cells that secrete hydrolytic enzymes to degrade the endosperm cell walls, allowing the radicle to emerge during germination. In contrast, cereal endosperm contains a core of mature starchy endosperm cells that are already dead before germination, and the enzymes that break down reserves are secreted by the cells in the aleurone layer (Leubner-Metzger et al., 1995; Nonogaki and Morohashi, 1996; Chen and Bradford, 2000; Brown and Lemmon, 2007). Secretory activity is therefore less likely in the starchy cereal endosperm, as has indeed been reported when cereal seeds are used for the expression of recombinant proteins. Recombinant proteins such as fungal phytase and the HIV-neutralizing antibody 2G12, carrying a signal peptide directing them to the endomembrane system but no further targeting signal, were shown to be transported to the PSV via the Golgi apparatus as evidenced by immunoelectron microscopy and glycan analysis in wheat, rice, and maize (Arcalis et al., 2004, 2010; Drakakaki et al., 2006; Peters et al., 2013). The same phytase protein, carrying the same targeting information, was secreted to the apoplast in leaves of transgenic rice plants (Drakakaki et al., 2006). These results suggest that the final destination for otherwise secreted proteins in the cereal endosperm is the PSV, although it is also possible that signal sequences are recognized and interpreted differently by cereal leaf mesophyll and seed endosperm cells.

### TRANSPORT BETWEEN THE ER AND PSV

The tracking of proteins in different storage compartments has shown that the PSVs in cereal endosperm cells are not simply post-Golgi compartments. Prolamins such as wheat glutenins, barley hordeins and oat avenins are initially deposited in ER-derived protein bodies, but later they are also found in the PSVs (Cameron-Mills and Wettstein, 1980; Lending et al., 1989; Rechinger et al., 1993; Shewry et al., 1995; Galili, 2004; Tosi et al., 2009; Ibl et al., 2014). How they get there is a matter of controversy, but it appears that both Golgi-dependent and Golgi-independent routes are involved. Levanony et al. (1992) initially suggested, based on a series of electron micrographs, that ER-derived prolamin bodies in wheat endosperm are eventually incorporated in the PSV through an autophagy-like process that bypasses the Golgi apparatus. This is supported by the presence of internal membranes in barley endosperm PSVs, including ER-derived membranes associated with individual and clustered protein bodies, suggesting that also hordein transport to the PSV at least partially bypasses the Golgi (Ibl et al., 2014). Ectopic zein bodies are also sequestered into PSVs in tobacco endosperm cells (Coleman et al., 2004). Reyes et al. (2011) proposed a Golgi-independent, autophagy-like route for the vacuolar delivery of zeins based on the presence of pre-vacuolar compartments that would sequester zein bodies and deliver them to the PSV in aleurone cells. Although the lipidation of ATG8 was observed, which is consistent with the macroautophagic mechanism (Klionsky et al., 2008; Rubinsztein et al., 2009; Chung et al., 2010), there was no subsequent vacuolar degradation of lipidated ATG8, indicating that the vacuolar delivery of zeins to the PSVs of maize aleurone cells does not use the typical ATG8-dependent macroautophagic process, but rather an atypical autophagy-like sequence of events (Aamodt et al., 2011). As an alternative to this model, Rogers (2011) proposed a mechanism by which membrane-containing vesicles bud from the ER as pre-vacuolar organelles that subsequently deliver materials into the PSV, which has been documented in dicots (Oufattole et al., 2005).

The direct route from the ER to PSVs in seeds involves a lot of traffic. Torres et al. (2001) reported the presence of the ER-resident molecular chaperone calreticulin in the rice endosperm PSV, providing evidence for a Golgi-independent pathway. Accordingly, KDEL-tagged recombinant proteins are often found at least partially in PSVs, e.g., a single chain antibody fragment (scFv) in rice (Torres et al., 2001) and HSA in wheat (Arcalis et al., 2004). The direct ER-to-PSV transport of recombinant proteins has also been reported in dicot seeds (Petruccelli et al., 2006; Floss et al., 2009; Loos et al., 2011a; Morandini et al., 2011; Arcalis et al., 2013). The endogenous KDEL-tagged proteinase sulfhydryl-endopeptidase (SH-EP) behaves in a similar manner in the cotyledons of germinating mung bean seeds (Toyooka et al., 2000; Okamoto et al., 2003), and storage protein precursors in pumpkin seeds are delivered to PSVs in precursor-accumulating (PAC) vesicles (Hara-Nishimura et al., 1998). PAC-like vesicles have also been described in rice endosperm by Takahashi et al. (2005). They are larger than the PAC vesicles in pumpkin seeds but they also arise directly from the ER and contain storage proteins destined for the PSV, such as glutelins and globulins (Takahashi et al., 2005). Viotti (2014) recently speculated that PAC vesicles may be the precursors of PSVs that slowly acquire their final size and identity via Golgi-mediated and post-Golgi-mediated transport, similar to the transition from pro-vacuoles to lytic vacuoles (Viotti et al., 2013; Viotti, 2014). Indeed, the main vacuolar proton pumps (H^+^-ATPase, VHA-a3 and H^+^-PPase AVP1) appear to follow a Golgi-independent route from the ER to the PSV in *Arabidopsis* seeds (Viotti et al., 2013), and alpha-TIP may follow the same, brefeldin A (BFA)-insensitive route. Considering this possibility, the trafficking of storage proteins and the relationships between the different storage compartments in cereal seeds could be reinterpreted, well in line with a model proposed earlier for barley aleurone PSVs (Bethke et al., 1998). In this model, it has been proposed that the ER membrane producing the neutral lipid may also serve as the site of storage protein accumulation and could mature into a PSV, supported by the observation that oleosomes forming on the surface of the ER are later found surrounding PSVs.

### DEVELOPMENTAL CHANGES OF PROTEIN TRAFFICKING ROUTES

A number of studies have suggested that the intracellular trafficking and distribution of storage proteins may change during seed development (Shy et al., 2001; Vitale and Hinz, 2005; Tosi et al., 2009). Accordingly, the fates of the recombinant glycoprotein phytase, and the endogenous vacuolar corn proteins legumin-1 (CL-1) and α-globulin (CAG), were found to change during maize endosperm development (Arcalis et al., 2010). All three proteins were found in the PSVs during early development as expected, but closer to maturity they were found at the periphery of the ER-derived zein bodies. In the case of recombinant phytase, the switch in localization was accompanied by a switch in the glycan profile, consistent with a stage-dependent protein trafficking behavior comprising a Golgi-dependent pathway to the PSVs in younger cells switching to the deposition in ER-associated compartments later. There was also a significant reduction in the number of PSVs during development (Arcalis et al., 2010), suggesting a net decrease in number or a change in appearance reflecting the different content. There was no significant decline in the number of Golgi organelles in developing maize, although in wheat the number of transcripts representing Golgi-associated proteins was shown to decline during seed maturation (Shy et al., 2001).

The endomembrane system of specialized and transient tissues such as endosperm should therefore not be considered as a static and rigid structure, but rather as a plastic and dynamic system that involves interactions between organelles and changes in protein trafficking to fulfill distinct developmental functions.

## SPATIOTEMPORAL MORPHOLOGICAL CHANGES OF STORAGE ORGANELLES IN CEREAL SEEDS

### ARCHITECTURAL CHANGES IN THE ENDOMEMBRANE SYSTEM DURING DEVELOPMENT AND GERMINATION

The massive reorganization of the endomembrane system during seed development involves significant morphological changes to the endosomal and storage organelles (Hoh et al., 1995; Wang et al., 2010; Ibl et al., 2014). The observation of *in vitro* transgenic maize endosperm cultures has shown that the ER changes from its typical reticulated distribution pattern into a more punctate architecture during the development of both starchy endosperm and aleurone cells, probably representing the formation of ER-derived protein bodies (Aamodt et al., 2011). In barley, the aleurone ER remains predominantly reticulated, whereas prominent protein bodies appear in the subaleurone and central starchy endosperm ERs (Ibl et al., 2014). In the germinating grain, the synthesis of secretory proteins in the living aleurone cells is accompanied by the proliferation of the ER to form stacks, an increase in the size and complexity of the Golgi apparatus, a reduction in the number of oleosomes and an increase in the number of glyoxysomes and mitochondria (reviewed by Fath et al., 2000).

**FIGURE 2:**
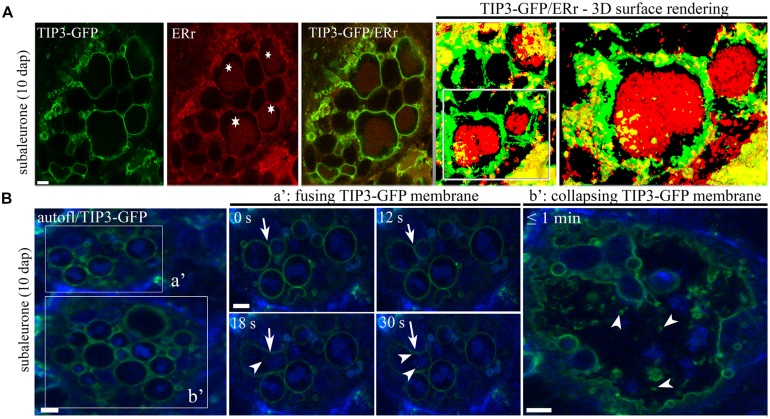
**TIP3-GFP-labeled PSVs contain protein bodies and are involved in fusion and rupture in barley endosperm. (A)** TIP3-GFP-labeled PSVs comprise large protein bodies stained with ER-Tracker^TM^ red (asterisks). Note the three-dimensional surface rendering (and the magnified inset) of 16 sections with a step size of 0.5 μm. **(B)** Live cell imaging of TIP3-GFP-labeled PSVs show fusion (a′) and collapse (b′) processes in the subaleurone at 10 DAP. Note the presence of TIP3-GFP-labeled vesicles (a′, arrowheads) after PSV fusion (a′, arrows). Images were acquired every 6 s. Scale equals 5 μm. Figure partially reproduced from Ibl et al. (2014).

Conventional studies of subcellular structures, intracellular pathways and protein storage organelles in cereal seeds involve the acquisition of static images based on immunofluorescence and electron microscopy (Cameron-Mills and Wettstein, 1980; Arcalis et al., 2004, 2010; Gubatz et al., 2007; Holding et al., 2007; Fukuda et al., 2013; Tian et al., 2013). This reveals little about the dynamic restructuring events during endosperm development, which have to be inferred from samples at different developmental stages. However, fluorescent membrane markers can be used to follow the dynamic morphological changes *in situ* by live cell imaging. This was reported in the context of barley endosperm development, comparing PSVs in the aleurone, subaleurone and central starchy endosperm layers (Ibl et al., 2014). Whereas the spherical PSVs in the aleurone remain constant, those in the subaleurone and central starchy endosperm cells undergo substantial but cell type-specific morphological changes. In both the subaleurone and central starchy endosperm cells, the PSVs initially appear as large compartments. In the subaleurone, they subsequently go through cycles of fusion and rupture, first allowing the protein bodies to form larger, composite aggregates within the vacuole, and finally producing unconfined protein bodies and ER-derived as well as TIP3-labeled membrane fragments (Figures **Figure 2A,B**). In contrast, PSVs in the starchy endosperm become smaller so that the protein bodies are tightly enclosed, and later in development some of the PSV membranes lose their integrity. This membrane degeneration may be induced by the desiccation process. The aleurone is protected against desiccation-induced injury (Stacy et al., 1999) but membrane dehydration may contribute to the dynamic behavior of membranes in the starchy endosperm due to the physical impact on membrane lipids, including demixing, fluid-to-gel phase transition, increasing cytosolic viscosity, protein denaturation, and membrane fusion (Bryant et al., 2001; Hoekstra et al., 2001). In rice endosperm, the synthesis of large amounts of disulfide-rich storage proteins during seed development is accompanied by the production of H_2_O_2_, which causes the peroxidation of membrane lipids (Onda et al., 2009). This promotes lipid chain fracture, which alters intrinsic membrane properties such as fluidity and permeability, finally leading to the leakage of small molecules and electrolytes (Sattler et al., 2006; Onda et al., 2009; Sharma et al., 2012). Notably, mutant rice seeds that cannot produce normal amounts of sulfhydryl groups generate less H_2_O_2_ and desiccate more slowly than wild-type seeds (Onda et al., 2009). The production of H_2_O_2_ in the ER of endosperm cells may also induce programmed cell death during seed desiccation and maturation (Onda, 2013).

### THE ALEURONE AS A STORAGE TISSUE

In addition to providing hydrolases for the breakdown of reserves in the starchy endosperm, the aleurone is also a storage tissue in its own right (Ritchie et al., 2000). Aleurone cells are filled with spherical PSVs that retain a constant appearance during endosperm development (Cameron-Mills and Wettstein, 1980; Bethke et al., 1996; Ibl et al., 2014). PSVs in the barley aleurone contain storage proteins, phytate (which is rich in phosphorus, potassium and magnesium) as well as storage carbohydrates (Jacobsen et al., 1971). *In situ* hybridization, RT-PCR and immunoblot analyses have shown that zein transcripts and proteins are present in the aleurone (Woo et al., 2001; Aamodt et al., 2011). Subcellular localization studies have revealed the presence of zeins, α-globulin and legumin-1 in maize aleurone cells at 18 and 22 days after pollination (DAP), predominantly as large inclusions within PSVs and small ER-derived protein bodies (Aamodt et al., 2011). As reported for the barley aleurone, PSVs and lipid bodies occupy most of the aleurone cellular volume in maize, along with multi-vesicular bodies (MVBs) and double-membrane autophagosome-like structures. Electron tomography has shown that aleurone cells at 22 DAP also contain one or more globoids (crystals of phytic acid salts) and a large system of intravacuolar membranes, possibly derived from the ER (Aamodt et al., 2011). Moreover, typical ER-resident proteins were detected in the PSVs (Aamodt et al., 2011). The dynamic analysis of the intravacuolar membrane system and the zein-rich inclusions in the maize aleurone revealed developmental changes in the PSV size, the total membrane surface area and the percentage corresponding to the intravacuolar membranes (Aamodt et al., 2011). The PSVs were smaller and more homogenous at 14 DAP compared to 22 DAP. The total membrane surface area declined by ∼50% during this interval, whereas zein-rich inclusions occupied almost 10-fold more of the PSV lumen at 22 DAP (Aamodt et al., 2011). The content and intravacuolar membrane system of maize aleurone PSVs therefore appears exquisitely dependent on the developmental stage.

### MORPHOLOGICAL CHANGES WITHIN THE ALEURONE DURING GERMINATION

Following imbibition, the embryo releases gibberellic acid (GA_3_), which diffuses into the aleurone layer and induces the highly differentiated and specialized aleurone cells to synthesize and secrete digestive enzymes that subsequently mobilize the insoluble reserves in the starchy endosperm (Ritchie et al., 2000). During this process, the aleurone cells break down their storage proteins and use the resulting amino acids to synthesize a spectrum of hydrolases (Jones and Jacobsen, 1991; Bethke et al., 1998). After a few days, the storage reserves of the endosperm are depleted and the aleurone cells die.

The process is accompanied by profound morphological changes in the endomembrane system of aleurone cells (Jacobsen et al., 1985). De-embryonated half-grains, isolated aleurone layers and aleurone protoplasts were used to study the responses of cereal aleurone cells to hormones and to understand molecular and cellular aspects of signaling and regulation in the aleurone (reviewed by Ritchie et al., 2000; Bethke and Jones, 2001).

Barley aleurone protoplasts treated with GA_3_ undergo dramatic morphological changes. Freshly isolated aleurone protoplasts contain many small PSVs, but 4 days of hormone treatment causes them to coalesce into one large central PSV (Jacobsen et al., 1985; Bush et al., 1986; Swanson and Jones, 1996; Fath et al., 1999). This GA_3_-dependent vacuolation process correlates strongly with the duration of GA_3_ treatment and is completed after 5 days of incubation (Bethke and Jones, 2001; Hwang et al., 2005). Granules within the vacuoles accumulate in larger numbers as the vacuoles increase in size. These morphological changes reflect a functional transition from nutrient-storing compartments to lytic organelles. Noninvasive measurements of the vacuolar pH in barley aleurone protoplasts showed that protoplasts respond to GA_3_, acidifying the vacuole lumen in the course of a few hours from pH 6.6 to 5.8 or below (Swanson and Jones, 1996; Swanson et al., 1998). The cells also showed an abrupt loss of membrane integrity (Fath et al., 2000). The prolonged incubation of aleurone protoplasts with GA_3_ was lethal.

Interestingly, gene expression profiling studies show that GA_3_ and abscisic acid (ABA) are both synthesized during seed maturation and germination and build a possible interaction network during seed germination (Sreenivasulu et al., 2006, 2008). GA_3_ and ABA signals also appear to influence the activity of a subset of stored proteases and the expression of further proteases and cell wall-loosening enzymes during grain maturation and germination. ABA antagonizes the events induced by GA_3_ in the aleurone at many levels, indicating a sensitive balance between these hormones involving a spatiotemporal interaction network of key regulators involved in storage organelle dynamics. Isolated aleurone protoplasts and layers have been used to study aleurone characteristics, especially the roles of GA_3_ and ABA in the regulation of vacuolar dynamics (Swanson and Jones, 1996; Swanson et al., 1998). Even if the same cellular events occur in imbibed whole grains and GA_3_-treated aleurone layers or protoplasts, the timing of events is faster in isolated aleurone layers and protoplasts and the model is simplified by the absence of interacting tissues and the application of GA or ABA, rather than their spatiotemporal ratios found *in vivo* (Bethke and Jones, 2001). The characteristics of aleurone protoplasts may also vary according to culture conditions (Jacobsen et al., 1985). These aspects emphasize the importance of confirming that results obtained using isolated cell cultures are also relevant in intact seeds.

### IDENTIFYING MOLECULAR REGULATORS OF STORAGE ORGANELLE BEHAVIOR

**FIGURE 3:**
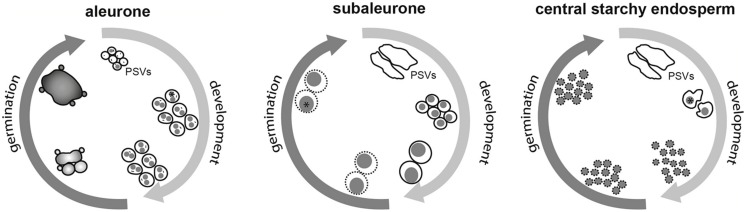
**Schematic overview of the spatiotemporally regulated dynamics of storage organelles in cereal seeds during development and germination.** The spherical PSVs in the aleurone remain constant throughout seed maturation. Note the presence of zein-rich protein inclusions (asterisk) and intravacuolar membranes (indicated as short black lines). Upon germination, GA_3_-dependent vacuolation results in the formation of one large central vacuole and secondary lytic vacuoles. The dramatic change from nutrient-storing compartments to lytic organelles is indicated by different shadings. In the subaleurone, large PSVs turn into smaller, spherical structures enclosing protein bodies (gray), followed by vacuolar fusion resulting in the formation of larger, composite protein bodies. The surrounding membranes gradually lose their integrity (indicated by broken lines). In the central starchy endosperm, the large PSVs containing protein bodies (gray) are shrinking. At later developmental stages, the protein bodies are tightly enclosed by PSV membranes, which degenerate during development, leaving protein bodies with no continuous PSV membrane or no membrane at all.

As discussed above, the dynamic behavior of storage organelles in cereal seeds strongly indicates the presence of a spatiotemporal regulatory network (**Figure 3**). Transcriptomics and proteomics have been instrumental in many areas of cereal research (reviewed by Pechanova et al., 2013). One opportunity to identify the molecular regulators of storage organelle-reshaping events is the transcriptional and/or proteomic analysis of developing and germinating seeds, to build an atlas of proteotypes. This has recently been reported for maize seeds, resulting in an atlas comprising ∼14,000 proteins (Walley et al., 2013). Total protein was extracted from dissected maize seeds at seven stages of development, including separated embryo, endosperm and aleurone/pericarp tissues. Normalized and averaged data showed that peptide homologs of *Arabidopsis* TIP3 were minimally expressed in the endosperm after 12 DAP, but increased in the endosperm and in the aleurone/pericarp tissue by 27 DAP. Similarly, the comparative transcriptional analysis of two tissue fractions during barley grain maturation, desiccation and germination (starchy endosperm/aleurone vs. embryo/scutellum) revealed that TIP3 expression increases steadily during maturation and declines steadily throughout germination (Sreenivasulu et al., 2008). Because the storage endosperm dies during late maturation, any increase in transcript abundance in this fraction during germination must depend on RNA that is newly synthesized in the aleurone (Sreenivasulu et al., 2008).

Precise sampling for the more specific spatial analysis of expressed proteins is challenging and a system that can differentiate between aleurone and starchy endosperm cells is necessary. Laser microdissection (LMD) overcomes the heterogeneous distribution of different tissues and cells by allowing spatially resolved sampling of individual cells, cell populations or tissues (Fang and Schneider, 2013). This approach was recently used for the tissue-specific transcriptomic analysis of barley seeds, allowing the detection of transcriptional networks in the context of endosperm development (Thiel et al., 2011). In combination with studies using transgenic lines with fluorescent marker proteins for cellular compartments, LMD-based transcriptomics provides a promising strategy to investigate the spatiotemporal regulation of storage organelle dynamics.

Recent developments in high-resolution, two-dimensional gel electrophoresis (2DE), multidimensional liquid chromatography and mass spectrometry (MS) have also helped to characterize the dynamic cereal seed proteome, e.g., in maize (Mechin et al., 2004) and barley (Finnie et al., 2004; Finnie and Svensson, 2009). Specialized extraction procedures allow the enrichment of enzymes and other proteins that control seed metabolism and storage product mobilization, in some cases allowing selective extraction from specific tissues and compartments such as the aleurone plasma membrane and starch granule proteomes in barley (Boren et al., 2004; Hynek et al., 2006). The resulting spatiotemporal profiling of the barley seed proteome during grain filling and germination allowed proteins to be assigned to distinct functional categories (Finnie and Svensson, 2003; Bonsager et al., 2007; Finnie et al., 2011). In maize, the availability of different mutants has facilitated proteomic analysis (Damerval and Devienne, 1993).

More insight into the dynamic nature of the seed proteome has been gained using comparative proteomics, and particularly quantitative proteomics. For example, comparative 2DE has been used to study the time course of protein mobilization during rice seed germination, revealing that germination causes the radical reprogramming of the proteome, not only by consuming protein reserves but also rebuilding defense pathways and inducing morphological changes (Yang et al., 2007; He et al., 2011a). Isobaric tags for relative and absolute quantification (iTRAQ) has been used to determine precise quantitative changes in the proteome during seed development (Lan et al., 2011; Owiti et al., 2011), and the same technique has been combined with multiple reaction monitoring (MRM) and gene ontology (GO) classification to profile the rice embryogenesis-dependent proteins, thus providing a dataset that could be used to investigate the molecular basis of rice embryogenesis (Zi et al., 2013).

## RECOMBINANT PROTEIN PRODUCTION IN CEREAL ENDOSPERM

### UNUSUAL TRAFFICKING BEHAVIOR OF RECOMBINANT PROTEINS

Protein trafficking and organelle reshaping can also be investigated by looking at the behavior of recombinant proteins, including storage proteins fused to fluorescent tags (Tosi et al., 2009; Saito et al., 2012; Shigemitsu et al., 2013; Wakasa and Takaiwa, 2013) or foreign proteins that have been produced in the context of molecular farming. Endogenous glycoproteins are rarely found in the endosperm (Woo et al., 2001) and are difficult to follow due to the lack of appropriate detection antibodies, so recombinant glycoproteins are particularly useful because they can be traced through intracellular pathways by immunolocalization and by characterizing their glycan modifications (Drakakaki et al., 2006; Arcalis et al., 2010).

A better understanding of the peculiar rules of protein trafficking in the cereal endosperm is also desirable for the production of valuable recombinant proteins such as pharmaceuticals, which need to be produced in a predictable and homogeneous form (Stoger et al., 2014). Targeting sequences that have been shown to work in vegetative plant organs do not always guarantee the predictable trafficking of recombinant proteins to a given compartment in cereal endosperm cells (Arcalis et al., 2004; Takaiwa et al., 2009; Wakasa and Takaiwa, 2013). For example, the same protein may be secreted from rice leaf cells, but sequestered in PSVs within the endosperm (Drakakaki et al., 2006). The addition of a KDEL signal usually targets proteins to the ER lumen in vegetative tissues, but in the endosperm the final destination appears to differ by species and protein, such as in the case of endogenous prolamins accumulating in the ER and ER-derived protein bodies in rice (Wakasa and Takaiwa, 2013) and maize (Rademacher et al., 2008), or in the PSVs of wheat endosperm (Arcalis et al., 2004). Storage protein fusions usually accumulate in predictable compartments, i.e., protein bodies or PSVs (Sugita et al., 2005; Wakasa and Takaiwa, 2013). Storage organelles are generally a desirable target site for recombinant proteins because they have evolved to facilitate stable protein accumulation and thus offer a protective environment for recombinant proteins, which can remain stable in dry cereal seeds at ambient temperatures for several years (Stoger et al., 2005b). Secondly, the accumulation of recombinant proteins into storage organelles can protect the protein not only from degradation within the plant cell but also beyond harvest. For example, prolamin bodies are highly resistant to gastrointestinal digestion *in vitro* and *in vivo*, resulting in the excretion of significant numbers of prolamin bodies, making them suitable for bioencapsulation (Tanaka et al., 1975; Ogawa et al., 1987). It should be borne in mind that the trafficking of recombinant proteins can change during seed development and affect the glycan profile as discussed above, so the harvesting time can significantly affect the characteristics of the purified protein (Arcalis et al., 2010).

Another factor that may impact not only the expression, but also the subcellular localisation and modification of recombinant proteins in the endosperm is the choice of promoter to drive transgene expression. The structure and arrangement of storage organelles varies according to the tissue layer within the endosperm (Tosi et al., 2009; Ibl et al., 2014), and endosperm-specific promoters also vary significantly in their spatiotemporal activity (Qu and Takaiwa, 2004), which should make possible to favor recombinant protein expression in cell types with a specific organelle architecture. Endosperm-specific promoters often have minimal or no activity in the aleurone (Lamacchia et al., 2001), so their use can be contrasted with constitutive or even aleurone-specific promoters such as the amylase promoter, which has been used to drive the expression of a recombinant enzyme during malting in barley (Nuutila et al., 1999). The secretory profile and *N*-glycoproteome differ markedly between the aleurone and the inner maturing endosperm (Barba-Espin et al., 2014). However, recombinant protein production during malting/germination is challenging because of the higher intrinsic proteolytic activity in the tissue at this stage, which affects the stability of the product.

Finally, the fate of a recombinant protein in developing seeds may occasionally be affected by interactions with endogenous storage proteins. Proteins with free cysteine residues and multimeric proteins that assemble via disulfide bonds (such as antibodies) are particularly prone to covalent interactions with prolamins. For example, free antibody chains interact with zeins (Peters et al., 2013), and a cysteine-rich peptide was shown to mediate protein retention in rice prolamin bodies (Takaiwa et al., 2009).

### THE BIOENCAPSULATION IN STORAGE ORGANELLES

Plant tissue can provide some protection from proteolytic enzymes in the gut, and the sequestration of recombinant proteins in rice protein bodies appears to extend the protection from digestive proteolysis following oral administration in an animal model. This was shown by directly comparing the digestibility of a model oral tolerogen from three sources: ER-derived protein bodies in rice endosperm, glutelin bodies (PSVs) in rice endosperm and a chemically synthesized peptide (Takagi et al., 2010). Both endosperm-derived peptides were more resistant to *in vitro* digestion with pepsin than the soluble form, but the prolamin bodies were more resistant than that from PSVs. Similar results were reported in feeding studies, indicating that bioencapsulated forms of the tolerogen significantly enhanced its immunological efficacy. The cholera toxin B subunit (CTB) also accumulated within protein bodies and the PSV in rice endosperm, and was similarly protected from *in vitro* pepsin degradation when fed to mice, allowing the induction of CTB-specific serum IgG and mucosal IgA antibodies (Nochi et al., 2007).

These studies suggest that protein storage organelles offer natural bioencapsulation strategies compatible with oral vaccines or prophylactic mucosal antibodies for passive immunization against gastro-intestinal disease. Oral vaccines must be protected from digestion to ensure sufficient amounts of the antigen reach the Peyer’s patches (lymphoid tissue in the ileum), which is necessary for the induction of an oral immune response. Storage proteins such as zeins have already been considered as *in vitro* protein encapsulation reagents for slow-release pharmaceuticals (Lai and Guo, 2011; Lau et al., 2013), thus naturally occurring protein bodies may provide the ideal encapsulation platform for recombinant pharmaceuticals produced in plants.

### ER STRESS AND NOVEL ER-DERIVED COMPARTMENTS

Some recombinant proteins have been shown to induce the formation of additional, prolamin-free ER-derived protein bodies, e.g., a modified house dust allergen and a modified birch pollen allergen expressed in rice seeds (Yang et al., 2012a; Wang et al., 2013). Similarly, human IL-10 expressed in rice seeds was found to be localized in ER-derived prolamin bodies and aberrant ER-derived compartments (Fujiwara et al., 2010; Yang et al., 2012b). The aberrant deposition of recombinant proteins in newly formed ER-derived structures has also been observed in other plant seeds, including a scFv-Fc antibody that was partially localized in ER-derived compartments delimited by ribosome-associated membranes in *Arabidopsis* seeds (Van Droogenbroeck et al., 2007). Similar observations have been reported for other scFv-Fc molecules with or without KDEL signals (Loos et al., 2011b). These novel ER-derived compartments may play a protective role in the host cell, and may be induced preferentially by aggregation-prone protein candidates. Although additional factors such as protein expression levels may also come into play, seeds could be more prone to the induction of ER-derived compartments because the mechanism that generates ER-derived protein bodies is already prevalent, especially in the endosperm.

Recombinant protein expression can sometimes elicit an ER stress response reflected by the coordinated expression of ER-resident molecular chaperones (Oono et al., 2010; Wakasa et al., 2012). This correlates with the appearance of distorted protein bodies at the microscopic level (Yang et al., 2012b; Wang et al., 2013). In some cases, there is also a specific grain phenotype at the macroscopic level, such as the opaque phenotype with floury and shrunken features reported in transgenic rice seeds (Oono et al., 2010), phenotypically similar to the maize *floury2* mutant that is also characterized by an ER stress response (Hunter et al., 2002). It is unclear whether seeds are more prone to ER stress induced by recombinant protein expression than vegetative tissues, perhaps due to the temporally confined high protein expression levels, or whether the stress symptoms are more obvious and therefore more frequently noticed in seeds due to the opaque phenotype.

### UNUSUAL *N*-GLYCAN STRUCTURES

*N*-glycan structures on recombinant proteins vary according to the final subcellular location and the trafficking route (Lerouge et al., 1998). The lack of secretion in cereal endosperm often results in a distinct lack of typical apoplast *N*-glycan structures such as GnGnXF, which are abundant in proteins expressed in leaves (reviewed in Samyn-Petit et al., 2003; Arcalis et al., 2013). Glycoproteins that have not passed through the Golgi apparatus typically bear oligomannosidic structures (high-mannose glycans) whereas those in post-Golgi locations tend to bear complex, xylosylated and fucosylated *N*-glycans. The ratio between these glycan classes sometimes correlates with the distribution of a protein between ER-derived protein bodies and PSVs, but this is not necessarily the case because the direct ER-to-PSV transport route discussed above may allow a proportion of proteins deposited in the PSV to bypass the Golgi apparatus completely. Interestingly, a number of recent studies involving the analysis of glycopeptides, rather than *N*-glycans digested off glycoproteins prior to analysis, have revealed significant proportions of aglycosylated seed-derived recombinant glycoproteins (Van Droogenbroeck et al., 2007). This may reflect the rapid production of high levels of the recombinant protein based on temporally confined extreme transcriptional and translational activities, but it may also indicate the limited capacity of the glycosylation machinery in maturing seeds. Under-glycosylation may also be linked to ER-stress and result in the formation of small, ER-derived organelles as discussed above.

Another, perhaps more puzzling phenomenon, is the abundance of trimmed *N*-glycan structures consisting of only a single GlcNAc residue, which may be useful for specialty applications but may not support the functionality of some recombinant proteins such as antibodies with effector functions (Umana et al., 1999; Schuster et al., 2007). These structures are often found on KDEL-tagged proteins, and most likely reflect the activity of Endo-β-*N*-acetylglucosaminidase (ENGase) on oligomannosidic *N*-glycan substrates (Fischl et al., 2011). Single GlcNAc residues have been detected on glycoproteins produced in maize, and may even represent the predominant glycoform suggesting a high level of ENGase) activity in cereal seeds (Rademacher et al., 2008; Ramessar et al., 2008a; Arcalis et al., 2010). Indeed, ENGase activity has been observed in cereal seeds (Vuylsteker et al., 2000), and free oligomannosidic glycan structures, which are released by ENGase activity, have been identified in seeds of diverse plant species (Kimura and Kitahara, 2000; Kimura et al., 2002) and a physiological role for them in plant development, fruit and seed maturation has been proposed (Priem and Gross, 1992; Kimura and Kitahara, 2000). However, *Arabidopsis* plants lacking ENGase activity showed no obvious morphological phenotype (Fischl et al., 2011; Kimura et al., 2011).

It is still unclear why ENGase, an enzyme with a presumed cytosolic localization (Suzuki et al., 2002; Fischl et al., 2011), can access substrates that accumulate within endomembrane organelles. Although we cannot completely exclude the possibility that glycan trimming occurs during extraction, our preliminary data instead support the *in vivo* action of the enzyme. In addition, based on the fact that endomembrane structures break down and compartments rapture, possibly in response to oxidative stress and desiccation (Ibl et al., 2014), combined with the incorporation of cytosolic material into the interior of vacuoles via atypical pre-vacuolar compartments during autophagy (Reyes et al., 2011), it could be speculated that these events might also allow ENGases to come into contact with glycoprotein substrates. Further investigations are required to determine whether this is the case, perhaps providing a better understanding of the physiological role of ENGases in plant seeds and increasing the spectrum of its endogenous substrates.

## CONCLUSION

Endosperm is a short-lived tissue that undergoes a rapid sequence of developmental changes, including extensive endomembrane system reorganization and rearrangement. The development and maturation of storage organelles is linked with storage protein synthesis and protein transport via several developmentally regulated trafficking routes. The investigation of spatially and temporally regulated gene expression will provide insight into the underlying molecular mechanisms, and transgenic cereal plants expressing fluorescent marker proteins combined with live cell imaging techniques allow the investigation of dynamic membrane reorganization events. The analysis of recombinant protein transport, deposition and glycosylation provides additional information about cargo trafficking to storage organelles. By understanding these processes, we will be able to control the production of recombinant proteins and design optimized production strategies for molecular farming that exploit the unique bioencapsulation properties of storage organelles.

## Conflict of Interest Statement

The authors declare that the research was conducted in the absence of any commercial or financial relationships that could be construed as a potential conflict of interest.
